# Selection of DNA Aptamers for Ovarian Cancer Biomarker CA125 Using One-Pot SELEX and High-Throughput Sequencing

**DOI:** 10.1155/2017/9879135

**Published:** 2017-02-09

**Authors:** Delia J. Scoville, Tae Kyu Brian Uhm, Jamie A. Shallcross, Rebecca J. Whelan

**Affiliations:** Department of Chemistry and Biochemistry, Oberlin College, 119 Woodland Street, Oberlin, OH 44074, USA

## Abstract

CA125 is a mucin glycoprotein whose concentration in serum correlates with a woman's risk of developing ovarian cancer and also indicates response to therapy in diagnosed patients. Accurate detection of this large, complex protein in patient samples is of great clinical relevance. We suggest that powerful new diagnostic tools may be enabled by the development of nucleic acid aptamers with affinity for CA125. Here, we report on our use of One-Pot SELEX to isolate single-stranded DNA aptamers with affinity for CA125, followed by high-throughput sequencing of the selected oligonucleotides. This data-rich approach, combined with bioinformatics tools, enabled the entire selection process to be characterized. Using fluorescence anisotropy and affinity probe capillary electrophoresis, the binding affinities of four aptamer candidates were evaluated. Two aptamers, CA125_1 and CA125_12, both without primers, were found to bind to clinically relevant concentrations of the protein target. Binding was differently influenced by the presence of Mg^2+^ ions, being required for binding of CA125_1 and abrogating binding of CA125_12. In conclusion, One-Pot SELEX was found to be a promising selection method that yielded DNA aptamers to a clinically important protein target.

## 1. Introduction

Ovarian cancer is diagnosed at local stage in only 15% of cases; five-year survival following this early diagnosis averages 92%. The majority of ovarian cancer patients are diagnosed with more advanced disease, for which the five-year survival rate is 46% [1]. Given the dramatic improvement in long-term survival rates enabled by early detection of ovarian cancer, the development of analytical tools to aid in screening and early diagnosis is of great importance [2].

Cancer Antigen 125 (CA125) is the clinical gold standard biomarker for ovarian cancer [3]. Serum levels of CA125 in ovarian cancer patients are regularly monitored, and a resurgence in CA125 correlates strongly with cancer recurrence [4–6]. Along with levels of the complementary biomarker HE4, serum levels of CA125 are considered in the Risk of Ovarian Malignancy Algorithm (ROMA), which is FDA-approved for the classification of women presenting with a pelvic mass into high- or low-risk categories [7, 8]. Another clinical use of CA125 is the Risk of Ovarian Cancer Algorithm (ROCA). ROCA predicts the probability of ovarian cancer based on serial measurements of serum CA125, exploiting the frequently observed phenomenon that ovarian cancer's initial development causes a change-point in these levels over a woman's individual baseline [9, 10]. The use of ROCA in ovarian cancer screening was assessed in the United Kingdom Collaborative Trial of Ovarian Cancer Screening (UKCTOCS), a randomized controlled trial that enrolled >200,000 postmenopausal women and followed them for 10 years [11]. The investigators of UKCTOCS report a significant reduction in mortality in the multimodal screening group—who underwent ROCA and sonography—relative to the no-screening control group, when women who entered the study with cancer are excluded from analysis [12]. These findings underscore the importance of excellent analytical assays for CA125 as part of the ongoing effort to refine existing ovarian cancer screening strategies.

CA125 is currently detected via double determinant immunoassay. Reliance on antibody-based detection is problematic for this biomarker, however, because of its heterogeneity. CA125 contains a variable number of repeat domains that are not identical and are recognized to differing extents by the most widely used classes of antibody [13]. The inability of available affinity reagents to recognize all repeat domains of CA125 suggests that existing clinical assays may significantly underestimate levels of this biomarker [3]. The desired clinical outcome of early detection may be enabled by the development of novel affinity probes that recognize CA125 in a manner different from existing antibodies, enabling more informative new detection methods.

As a first step towards the development of assays for ovarian cancer that do not rely on antibodies, we have selected single-stranded DNA aptamers with affinity for CA125. Aptamers are functional oligonucleotides selected through an in vitro evolution process to possess a binding or catalytic property of interest; the aptamer selection process is referred to as SELEX, an acronym for Systematic Evolution of Ligands by EXponential enrichment [14, 15]. SELEX typically occurs over multiple cycles, or rounds, each involving the incubation of candidate aptamer molecules with a target of interest; the partitioning of good binding oligonucleotides from those displaying weaker binding; and the amplification of good binders via polymerase chain reaction. At the conclusion of SELEX, the enriched oligonucleotide pool is subject to some form of sequence determination, typically via cloning into a bacterial expression system followed by Sanger sequencing.

Herein, we report on our use of One-Pot SELEX, in which selection and amplification of candidate oligonucleotides occur in a single container [16], to identify aptamers with affinity for CA125. The One-Pot approach minimizes transfer steps, mitigating both contamination and the loss of good binders. We conclude the aptamer selection process by sequencing DNA from each round on an Illumina platform. This data-rich approach enables the entire selection process, and not merely its endpoint, to be characterized. We have recently reported on a bioinformatics pipeline that enabled the identification of DNA aptamers with affinity for the ovarian cancer biomarker HE4 [2]. This pipeline has now been used to mine another large dataset of sequence information to identify aptamers for another important clinical target.

## 2. Experimental Section

### 2.1. Reagents

Oligonucleotides—including unselected DNA library, PCR, and sequencing primers, and labeled aptamers for in vitro testing—were purchased from Integrated DNA Technologies (Coralville, IA). The sequences, previously reported by Bowser and coworkers [17] were forward primer 5′-FAM-AGC AGC ACA GAG GTC AGA TG-3′, reverse primer 5′-biotin-TTC ACG GTA GCA CGC ATA GG-3′, ssDNA library 5′-FAM-AGC AGC ACA GAG GTC AGA TG (N)_25_ CCT ATG CGT GCT ACC GTG AA-3′. Hand-mixing was used in the synthesis of the DNA library's random region to mitigate differential coupling efficiency of nucleobases that can bias syntheses using machine mixing. All oligonucleotides were reconstituted in TE buffer (10 mM Tris, 0.1 mM EDTA, pH 8.0) to 100 *μ*M. Nuclease-free water, 25 mM MgCl_2_, 5.0 U/*μ*L Taq polymerase (for PCR), and Blue/Orange 6x loading dye (for gel loading) were purchased from Promega (Madison, WI). Deoxyribonucleotide triphosphates (dNTPs, 10 mM stock) were obtained from QIAGEN, Inc. (Valencia, CA). NuSieve GTG agarose was purchased from Cambrex BioScience (Rockland, ME). Selection and PCR amplification were performed in 0.5 mL thin-walled polypropylene tubes (Eppendorf North America, Hauppauge, NY). Sodium fluorescein was purchased from Sigma Aldrich (Saint Louis, MO). Tween-20 was purchased from Tokyo Chemical Industry (Portland, OR). CA125 from human ascites fluid (>95% purity) was purchased from Fitzgerald Industries International (Acton, MA) and supplied as a liquid in PBS buffer, pH 7.2, containing 3% sucrose and 0.05% sodium azide. Cancer antigen CA125 enzyme immunoassay test kit (ELISA kit) was purchased from BioCheck (Foster City, CA).

All buffers were prepared using 18.2 MΩ-cm water from a Milli-Q water purification system (Millipore Corp., Bedford, MA). Preweighed salts for preparing Tris-Glycine and Phosphate Buffered Saline were from Thermo Scientific (Rockford, IL). Protein incubating buffer contained 0.01 M NaPO_3_ and 0.15 M NaCl at pH 7.2. Protein washing buffer consisted of 0.1 M NaPO_3_, 0.15 M NaCl, and 0.05% v/v Tween-20 at pH 7.2 (PBT buffer). DNA binding buffer consisted of 25 mM Tris, 192 mM glycine, 5 mM KH_2_PO_4_, and 1 mM MgCl_2_ (TGKM buffer) adjusted to pH 8.3 with 1 M NaOH solution. CE separation buffer contained 25 mM Tris, 192 mM glycine, and 5 mM KH_2_PO_4_, pH 8.3 (TGK) prepared from Thermo Scientific Tris-Glycine powder and KH_2_PO_4_ from Mallinckrodt Chemical Works (St. Louis, MO). 10x single-stranding buffer consisted of 100 mM Tris, 20 M NaCl, and 10 mM EDTA (10x TNE buffer) at pH 7.5. Streptavidin agarose was purchased from Thermo Scientific Pierce Biotechnology Inc. (Rockford, IL). BioRad columns were purchased from BioRad Technologies (Hercules, CA). Binding and Washing (B&W) buffer (10 mM Tris, 2 mM NaCl, and 1 mM EDTA at pH 7.6, 2x concentration) was made from NaCl purchased from VWR (Bridgeport, NJ). All buffers were filtered through a 0.45 *μ*m nylon filter prior to use.

### 2.2. In-Tube ELISA

A modified ELISA was performed to identify optimal conditions for immobilizing CA125 in PCR tubes. One tube (“Overnight”) was incubated with 100 *μ*L of 8000 U/mL CA125 overnight at 4°C. A second tube (“SpeedVac”) contained 100 *μ*L of 8000 U/mL CA125; solvent was evaporated on a Savant DNA 120 OP SpeedVac Concentrator (Thermo Scientific, Asheville, NC) for four hours with no heating. Tubes were washed 4x with 200 *μ*L PBT and dried, after which the recommended protocol from the CA125 ELISA kit was followed; however, rather than using the CA125-coated microplate provided with the kit, the PCR tubes were used. Along with ELISA kit reagents, 30 *μ*L of 8000 U/mL CA125 was added to the positive control tube. The negative control tube was incubated with PBS overnight.

### 2.3. One-Pot SELEX

100 *μ*L of CA125 (8000 U/mL in 1x PBS) were added to five 0.5 mL PCR tubes. Three tubes were used for aptamer selection, one tube was used for PCR cycle determination, and one tube served as a negative control. Solvent was evaporated on a SpeedVac Concentrator for 4 hours with no heat. Tubes were then washed 4x with 200 *μ*L PBT. 25 N DNA library in DNA binding buffer was heat cycled at 95°C for 3 minutes and slowly cooled on ice prior to selection. In the initial SELEX round, 50 *μ*L of 1 *μ*M DNA library (diluted using DNA binding buffer) was added to the tubes and incubated for 1 hour at room temperature. The volume of DNA solution was less than that of the CA125 solution to minimize nonspecific interaction of DNA and the tube surface; for the same reason care was taken to pipet DNA solution directly into the center of the tube. 50 *μ*L of DNA binding buffer was added to negative control tube. DNA was removed from the tubes, and the tubes were washed with 200 *μ*L PBT. The number of washes at this step provided a means of increasing the selective pressure; over the course of four selection rounds, 6, 9, 12, and 15 wash steps were used. Vacuum was used to completely dry the tubes. In subsequent selection rounds, the highest available ssDNA concentration was used (typically less than 1 *μ*M). DNA concentration was determined by absorbance at 260 nm on a NanoDrop 2000 UV-vis spectrophotometer.

### 2.4. PCR Amplification

A PCR mastermix (600 *μ*L total volume) was prepared from 375 *μ*L nuclease-free water, 12 *μ*L 10 mM dNTPs, 9 *μ*L 10 *μ*M FAM-labeled primer, 9 *μ*L 10 *μ*M biotinylated primer, 72 *μ*L 25 mM MgCl_2_, 120 *μ*L 5x colorless PCR buffer, and 3 *μ*L 5 U/*μ*L Taq polymerase. 100 *μ*L of this mixture was aliquoted to the five 0.5 mL PCR tubes that underwent selection; these tubes were stored in the freezer while the optimal PCR cycle number was determined. 49 *μ*L of the mixture was added to separate PCR tube with 1 *μ*L of 100 nM library DNA as a PCR positive control. 49 *μ*L of the mixture was added to another PCR tube with 1 *μ*L of nuclease-free water as a PCR negative control. The cycle determination tube, One-Pot negative control tube, PCR positive control tube, and PCR negative control tube were placed in Mastercycler Personal (Eppendorf, Hamburg, Germany). All tubes were heated to 95°C for 10 minutes. 16 to 24 cycles of PCR were performed, each involving denaturation at 95°C for 30 seconds, primer annealing at 55°C for 15 seconds, and extension at 72°C for 15 seconds. Sample (10 *μ*L) was removed from the thermocycler after each round, mixed with gel loading dye, and stored on ice until analyzed. Electrophoresis was performed in a 4% agarose gel to determine the optimal cycle for amplification and to check for contamination in the two negative controls (the FAM primer enabled visualizing of bands without the use of ethidium bromide staining). Three selection tubes were stored at −20°C during cycle determination. The contents of these tubes were amplified using the optimal cycle number, with the addition of a final extension at 72°C for 5 minutes. The contents of the three selection tubes were pooled (total volume = 300 *μ*L).

### 2.5. Single-Stranding and Ethanol Precipitation

The dsDNA produced via PCR was converted to ssDNA using a streptavidin column to capture dsDNA through the biotinylated (undesired) strand, followed by incubation in alkaline solution to denature the two DNA strands. 300 *μ*L streptavidin agarose was loaded into a BioRad column and washed 4x with 500 *μ*L 2x single-stranding buffer. 300 *μ*L of PCR product and 300 *μ*L of 2x single-stranding buffer were added to the column and incubated for 30 minutes at room temperature with occasional vortexing. The column was allowed to drain completely and was washed 4x with 500 *μ*L 1x single-stranding buffer, followed by a final wash with 500 *μ*L nuclease-free water. 200 *μ*L of ~0.1 M NaOH was added to the column, and the column was incubated for 10 minutes at 37°C. Column contents, containing the fluorescein-labeled ssDNA of interest, were eluted into a 0.5 mL Eppendorf tube containing acetate buffer made from 116 *μ*L 0.27 M acetic acid and 35 *μ*L 3 M sodium acetate. The elution process was repeated with a second portion of NaOH solution; the products were collected into a second Eppendorf tube. 1000 *μ*L of 100% ethanol was added to the tubes, which were incubated in an ice bath for 1 hour or stored in a −20°C freezer overnight to precipitate ssDNA. Tubes were centrifuged at 14,000 rpm, 4°C, for 1 hour to pellet ssDNA. All but 50 *μ*L of solvent was aspirated from above the pellet, which was not always visible. The pellet was resuspended in 70% ethanol and tubes were again centrifuged for 15 minutes at 14,000 rpm and 4°C. This step was repeated, after which the DNA was dried on a SpeedVac for 15 minutes under high heat. 30 *μ*L water or DNA binding buffer was added to the dried tubes to reconstitute the DNA for the next round of selection or for sequencing. Samples were stored at −20°C until further use.

### 2.6. Sequencing and Bioinformatics

The process of sequencing and bioinformatics has been previously reported [2]. Briefly, after aptamer selection was complete, DNA collected from each round was amplified using Illumina sequencing primers. Archived DNA from each round was diluted to 100 nM using ultrapure water. Each sample was assigned a unique reverse primer containing the index used for barcoding. Samples and controls were amplified by PCR using an optimized cycle number. PCR products were imaged on a 3% agarose gel containing 1 *μ*g/mL ethidium bromide to confirm yield and the absence of contaminants or byproducts. Samples were sequenced at the University of Wisconsin Biotechnology Center DNA Sequencing Facility.

Bioinformatic screening of the sequenced DNA used a data pipeline based on freely available software, with the exception of enrichment analysis, which used a locally written Python program. This program (enrichment.py) has been made available on GitHub at https://github.com/rebeccawhelan/PythonEnrichment. After preliminary analysis of FastQC files to ensure the sequencing was successful, data were read into a Biopieces pipeline using read_fastqc. Each line of sequence data, corresponding to one aptamer candidate, was then modified by the removal of the (conserved) primer regions; sequences with length other than 25 ± 2 were discarded and sequences were counted and ranked based on abundance. Next, the processed data were taken through enrichment analysis, to determine fold enrichment for sequences across rounds of selection. Fold enrichment has been shown to be a more reliable indicator of binding affinity than read counts [18]. A composite score (compScore) was then calculated using

(1) $$\begin{array}{c} compScore=\log\left(\;\left[\;\overset{n}{\underset{i=1}{\prod }}\frac{R_{i}}{R_{0}}\;\right]\frac{R_{n}}{R_{0}}\;\right). \end{array}$$

The compScore is therefore the log10 of the product of each round's enrichment, with double weight given to the final round, in which the selective pressure was the most stringent. Using CD-HIT-EST [19], the top 1000 most enriched sequences from each round were clustered by sequence homology to determine possible emergent motifs. Sequences were clustered with their primers attached to a sequence identity threshold of 0.8 and assigned to clusters by the highest identity across all clusters.

### 2.7. Fluorescence Anisotropy

Fluorescence anisotropy was measured using a SpectraMax M5 multimode plate reader with polarizing optics (Molecular Devices, Sunnyvale, CA). Tested aptamers were ordered from Integrated DNA Technologies, Inc. (Coralville, IA) with a 5′ TEX615 (Texas Red) fluorophore. 100 nM DNA aptamer in buffer (TGKM) was heated to 97°C for 3 minutes, cooled on ice for 5 minutes, and then allowed to warm to room temperature. Heat-cycled DNA solution was combined with CA125 at a range of final concentrations from 0 U/mL to 2000 U/mL in the presence of 0.13 mg/mL bovine serum albumin (BSA) to prevent adsorption to the container walls. PBS buffer was added to achieve the desired final sample volume. After incubating for 90 minutes in the dark at room temperature, samples were loaded in duplicate or quadruplicate (75 *μ*L/well) into a 96-well Fluotrac 200 black immunology plate (USA Scientific, Ocala, FL) and analyzed in the SpectraMax, with temperature held at 22°C. The *λ*_ex_ for fluorescence anisotropy was 585 nm, *λ*_em_ was 635 nm, and the wavelength cut-off was 610 nm. Raw data (fluorescence emission parallel and perpendicular to the excitation) were blank-corrected before the anisotropy values were calculated. Data were fit with an isotherm function:

(2) $$\begin{array}{c} r-r_{0}=\frac{\left(constant\times T\right)}{\left(K_{d}+T\right)}, \end{array}$$

where *T* is protein concentration (varied), *A* is aptamer concentration (constant), *r* is the anisotropy measured in the presence of protein, and *r*_0_ is the anisotropy in the absence of protein, using IgorPro (v. 6.12) graphing software. To test the prediction that magnesium ion affected the affinity of aptamers for CA125, the assay described above was repeated, but with PBS used instead of TGKM at the heat-cycling step; in these assays samples were incubated for 24 hours at 4°C.

### 2.8. Affinity Probe Capillary Electrophoresis

Affinity probe capillary electrophoresis affinity assays were performed using a Beckman P/ACE MDQ (Beckman Coulter, Fullerton, CA) equipped with an argon-ion laser. An unmodified fused silica capillary (Polymicro Technologies, Phoenix, AZ; ID = 50 *μ*m, OD = 360 *μ*m, total length = 49.5 cm, and length from inlet to detector = 10.5 cm using reverse injection and negative polarity) was held at 25°C. Each sample contained 20 nM FAM-labeled aptamer (synthesized as a 25 mer sequence without primer regions), 20 nM fluorescein (internal standard), and 0.2 mg/mL BSA. TGKM was used both as the diluent in sample preparation and as the electrophoresis buffer for experiments with CA125_1_NP; TGK was used as the electrophoresis buffer for CA125_12_NP. To prepare samples, a bulk solution of aptamer in either TE or TGKM was heated to 97°C for 3 min and then cooled on ice. Fluorescein and BSA were then added, and the solution was distributed over an appropriate number of sample tubes. Finally, CA125 was added to a final concentration ranging from 0 U/mL to 2000 U/mL. The volume of protein plus protein buffer (PBS) was constant in all samples (4 *μ*L). Samples were incubated in the dark for 90 min. Pressure injection (0.3 psi, 5 s) was used to introduce sample onto the capillary; 0.5 psi pressure was applied to drive the sample plug past the uncooled region [20]. Separation was achieved by the application (in negative polarity) of 30 kV and 0.3 psi pressure for CA125_1_NP. Separation for CA125_12_NP was conducted at 20 kV with no applied pressure. Run time was 5 min. The fluorescence was excited at 488 nm and detected at 520 nm. Peak heights were determined by the instrument control software (32 Karat). The change in the size of the free DNA aptamer peak, relative to the internal standard, was used to indicate the complex formation between aptamer and protein. Data were fit with an isotherm equation:

(3) $$\begin{array}{c} Ratioed\;\;peak\;\;height=\frac{\left(constant\times T\right)}{\left(K_{d}+T\right)}, \end{array}$$

where *T* is protein concentration (varied) and *A* is aptamer concentration (constant), using IgorPro (v. 6.12) graphing software.

## 3. Results and Discussion

A single-stranded DNA library with *N* = 25 random region was used as the input to the selection process because it provided a good balance of sequence diversity, coverage, and computational tractability. Assuming that each base is equally likely to appear at each position in the random region, there are 4^25^ (~1 × 10^15^) possible sequences in such a library. In our selection, we used 50 pmol (~3 × 10^13^ molecules) of DNA as the initial input, giving any individual sequence an expected abundance of 0.03 (a library with *N* = 23 would give an expected abundance of 1). Using a longer random region would result in lower coverage of sequence space that could result in the loss of useful motifs, whereas a shorter random region might lack the complexity to form relevant secondary and tertiary structures involved in target binding.

**Figure 1:**
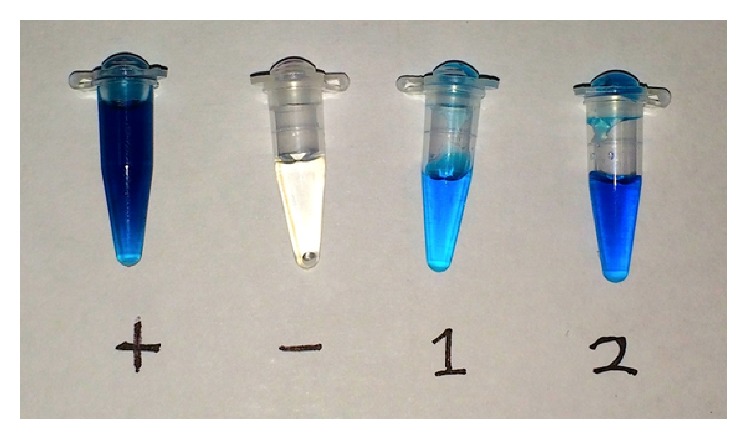
In-tube ELISA tests in CA125-coated PCR tubes. Positive control (+) contains 30 *μ*L of 8000 U/mL CA125. Negative control (−) was incubated with PBS overnight. Tube 1 was incubated with 30 *μ*L of 8000 U/mL CA125 overnight at 4°C. Tube 2 contained 30 *μ*L of 8000 U/mL CA125 and was dried without heating on a SpeedVac for 4 hr. Image taken with an iPhone 5s.

Figure 1 shows the results of an in-tube ELISA used to evaluate different methods for adsorbing CA125 within PCR tubes. Based on a qualitative assessment of color change, both overnight incubation at 4°C and 4 hr of solvent evaporation on a SpeedVac resulted in the adsorption of immunologically active CA125 to the walls of PCR tubes. SpeedVac treatment was observed to cause greater color change for the same input concentration of CA125 and assay reagents and took less time to be completed, so this approach was used in One-Pot SELEX.

**Figure 2:**
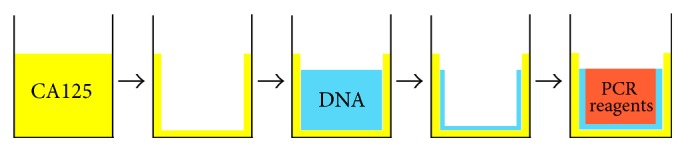
Schematic representation of One-Pot SELEX. All steps occur in a single PCR tube. First the protein target (CA125) is allowed to adsorb to the inner surface of the tube. Multiple rinsing steps remove all proteins that are not strongly adsorbed. Then ssDNA—either unselected library or DNA selected in the previous SELEX round—is added and allowed to equilibrate with adsorbed protein. Vigorous rinsing retains only those DNA molecules that bind the protein target with high affinity. These DNA sequences are amplified via PCR in the final step.

One-Pot SELEX is represented schematically in Figure 2. In One-Pot SELEX, selection and amplification occur in the same PCR tube. This approach distinguishes One-Pot from SELEX modes in which other means of separation (nitrocellulose filtration, column chromatography, capillary, or chip electrophoresis) enable the partitioning of high-affinity binding aptamer candidates from those candidates that do not bind target. After incubation of the aptamer candidates with the CA125 adsorbed in the tube, the tube was rinsed with buffer to remove weakly bound and nonspecifically bound DNA. Iteratively increasing the number of wash steps (from 6 to 15 over the course of four selection rounds) applied greater selective pressure and forced the population of DNA to converge on higher affinity binders.

**Figure 3:**
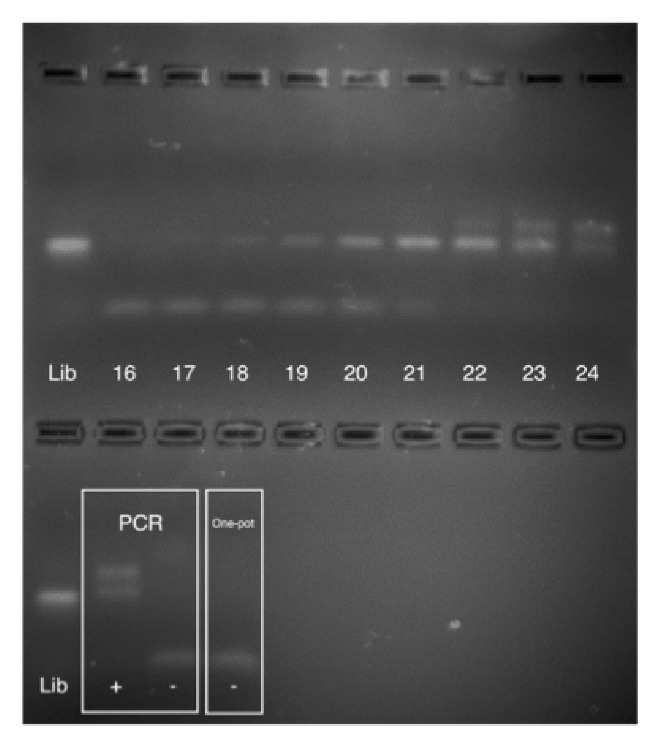
Representative agarose gel image showing the results of cycle determination, PCR positive and negative controls, and One-Pot negative control. The optimal number of PCR cycles is determined by removing reaction tubes from the thermocycler at regular intervals and electrophoresing the reaction mixtures together on a gel. In this case, 21 PCR cycles gave desired PCR product with minimal formation of more slowly migrating byproducts. The gel image also shows that positive and negative PCR controls give the correct outcome (product and no product, respectively). Finally, the One-Pot negative control shows that tubes to which no DNA was added do not give any PCR product, as expected.

Our group and others have documented that PCR amplification of the random DNA libraries used in aptamer selection is subject to undesirable side-reactions [2, 21]. Specifically, with increasing cycles of PCR, the dsDNA product of interest initially increases in abundance but is consumed in a side reaction yielding byproducts that migrate more slowly under electrophoresis in agarose gels. To minimize the negative impact of byproduct formation, we optimized the number of PCR cycles after each SELEX round. One reaction tube was subject to PCR, and sample was removed from this tube at regular intervals. Samples were electrophoresed together on a 4% agarose gel and imaged on a gel box with ethidium bromide filters.  Figure 3 shows a typical gel image. In this experiment, 21 cycles of PCR were found to give the highest yield of the desired product with minimal byproduct formation. The image also shows expected outcomes for the positive and negative PCR controls and for the One-Pot control, in which CA125 was immobilized in the tube but DNA-free buffer was added during the incubation step.

**Figure 4:**
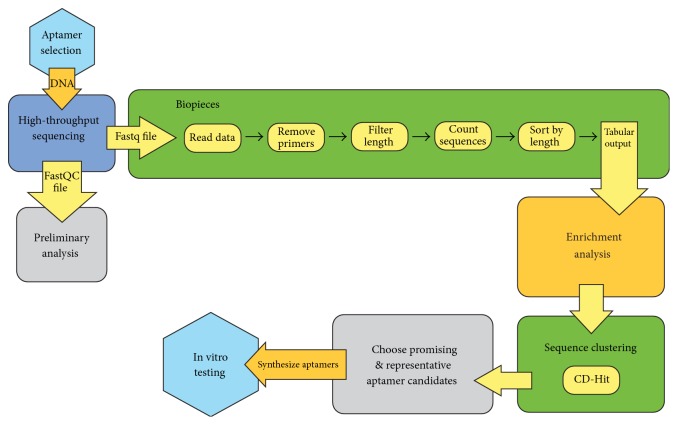
Flowchart showing the steps involved in analyzing high-throughput sequence data collected after a SELEX experiment. A series of sequence modification and counting steps is accomplished using Biopieces. Once tabulated based on abundance, the sequence information is analyzed using a locally written enrichment analysis script. CD-HIT is used to identify cluster sequences, and aptamers are chosen for characterization in vitro based on enrichment in response to the selective pressures of SELEX, their placement in a large sequence cluster, or both.

The bioinformatics pipeline used after the completion of One-Pot SELEX is represented in Figure 4. This pipeline is an improved version of a similar computational process that we developed to identify DNA aptamers with affinity for ovarian cancer biomarker HE4 in a previous study [2]. DNA archived after each selection round was sequenced on an Illumina platform. This approach yields more reads (max = 10.8 × 10^6^, min = 4.1 × 10^6^, average = 6.6 × 10^6^) than would be achievable with a clone-and-sequence method, which typically yields 10–100 sequences.  Table 1 contains information about the resulting sequence data. We note two attributes in this table. First, the—ostensibly random—unselected library contains duplicate sequences. We previously reported such sequence duplication in unselected libraries [2] and attribute it to errors in synthesis and sequencing. In addition, the percent duplicated sequences increase during selection, from 15.24% in the unselected library to 85.04% in DNA selected in Round 4. Sequence duplication increases for both CA125-binding DNA (designated by + in Table 1) and unbound DNA (designated by –). However, the rate of increase is greater in the population of CA125 binders. We interpret these data to mean that the selective pressures have substantially altered the ssDNA population, and that the effect occurred first in the population of DNA that bound to the protein target. Considering that the input for each selection round was the binding DNA from the previous round, it is reasonable that sequence duplication in the positive and negative selection rounds should proceed as observed.

The data reveal synthesis bias in the unselected DNA library. Ideally, the “random” region of the unselected library will contain an equal proportion of the four nucleobases; in our library the random region contained 24.3 ± 0.5% G; 23.8 ± 0.2% A; 27.4 ± 0.3% T; 24.4 ± 0.4% C. Although this library—prepared with hand-mixing—does not adhere to the ideal base ratio, it is less biased than other libraries we have examined that were prepared by machine mixing (data not shown). Hand-mixing is therefore advisable in the preparation of libraries for aptamer selection.

Four promising aptamer sequences were chosen for testing in vitro; these sequences are shown in Table 2. Candidates were chosen that were substantially enriched during the selection process, belonged to secondary structure clusters, or both. Sequence CA125_1 was the most enriched sequence by the final round of selection (Round 4). Sequence CA125_2 has a strong composite score and a steady increase over all selection rounds. Unlike the other sequences we tested, CA125_2 is 26 bases long; our bioinformatics analysis was designed to include all sequences of length 25 ± 2. This 26 mer sequence is most likely an artifact of PCR or synthesis error, but we chose to characterize its affinity for CA125 because of its high degree of enrichment. Sequence CA125_3 is representative of the sequence cluster that seems dominant in the most highly enriched selection round. Finally, sequence CA125_12 is representative of the highest-scoring cluster.

**Figure 5:**
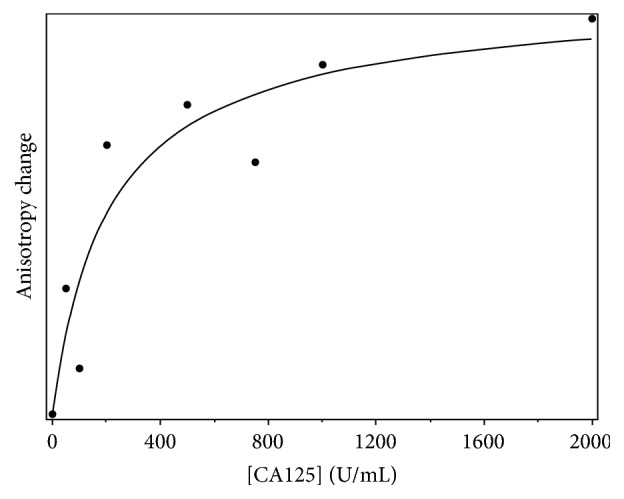
Binding isotherm determined from fluorescence anisotropy of samples containing 100 nM Texas Red-labeled aptamer CA125_1 and varying concentrations of CA125. Fluorescence was excited with polarized light at 585 nm; emission at 635 nm was collected along optical paths parallel and perpendicular to the excitation light and used to calculate anisotropy.

Two independent analytical methods—fluorescence anisotropy (FA) and affinity probe capillary electrophoresis (APCE)—were used to evaluate the binding affinity of these four aptamer candidates for CA125. When the *N* = 25 (or *N* = 26, in the case of CA125_3) random regions of the aptamer candidates were assayed, two of them—CA125_1 and CA125_12—displayed concentration-dependent binding to CA125.  Figure 5 shows a binding isotherm for CA125_1, resulting from monitoring FA change as CA125 concentration was increased from 1 to 2000 U/mL. On the *y*-axis is plotted the change in anisotropy relative to the free aptamer in the absence of protein, an indication of complex formation; CA125 concentration is the independent variable. The data are well fit by the binding isotherm equation, yielding an apparent *K*_*d*_ of 207 ± 109 U/mL. Binding required that CA125_1 be heat cycled in the presence of 5 mM magnesium ion prior to incubation with protein target; CA125_1 heat cycled in buffer depleted of magnesium ion did not display concentration-dependent binding to CA125. Data from FA assays are supported by APCE assays, which yield an apparent *K*_*d*_ of 80. ± 38 U/mL. Aptamer candidate CA125_12 was found to display concentration-dependent binding to CA125 as well, with *K*_*d*_ values of 118 ± 123 U/mL and 131 ± 93 U/mL determined by FA and APCE, respectively. In contrast with CA125_1, which required heat cycling in the presence of magnesium ion for binding, CA125_12 bound to CA125 only when heat cycled in PBS or TE without magnesium ion; heat cycling in the presence of magnesium ion substantially reduced the binding affinity of CA125_12. The variation in *K*_*d*_ values reported here is within the range observed by our lab and others for DNA aptamer-protein pairs characterized by FA and APCE [2, 17]. We chose to use a simplified form of the isotherm equation because the generalized form requires the addition of terms with dissimilar units: U/mL for CA125 concentration and nM for aptamer concentration [22].

The units in which we have chosen to report *K*_*d*_ values—U/mL—merit discussion. Because of the long-documented heterogeneity of CA125 [3], this protein does not have a well-defined value for molar mass. For clinical and research applications, concentrations of CA125 are always reported in U/mL, a unit that derives from the immunoassay that is the standard for clinical detection. Although this unit is not familiar to chemists and biologists, reporting CA125 in this manner is standard practice for ovarian cancer researchers and clinicians. The current clinical cut-off for CA125 is 35 U/mL, and commercially available research grade immunoassay kits typically have a dynamic range up to 200 U/mL. Our DNA aptamers therefore have an apparent affinity at a clinically relevant concentration.

The other two aptamer candidates (CA125_2 and CA125_3) did not display binding to CA125 when examined as random region alone. The reason for the absence of positive binding as indicated by these two in vitro assays is not clear. We note that the two successful aptamer candidates (CA125_1 and CA125_12) were, respectively, the most enriched in response to selective pressure and representative of the highest-scoring cluster. The other candidates had less favorable enrichment metrics, as determined by bioinformatics analysis. In addition, we note that whereas the aptamer candidates that displayed target binding contained all of the four nucleobases, the two unsuccessful aptamer candidates tested in vitro contain no G and have a percentage of T higher than the expected percentage (25%).  Table 3 shows the base composition of the four aptamer candidates we tested in vitro. Those that did not display binding (CA125_2 and CA125_3) contain 15% and 12%, respectively, of G + C whereas those that displayed binding (CA125_1 and CA125_12) both contained 40% G + C (random distribution would predict that each sequence would contain 50% G + C). Given the stronger intramolecular forces associated with G-C base-pairing over A-T base-pairing, this difference in nucleobase content may be significant in the observed binding to the protein target. Finally, assays involving aptamer candidates bearing the PCR primer-binding regions did not show evidence of concentration-dependent complex formation as the concentration of CA125 was increased (data not shown).

## 4. Conclusions

In its original applications, One-Pot SELEX successfully identified aptamers with affinity for antibodies [16, 23]. In this report we have shown its use for selection of aptamers recognizing CA125, a mucin protein that is the gold standard biomarker for ovarian cancer. Given the widespread importance of mucins in many types of cancer [24, 25], this relatively simple and efficient process might enable the selection of clinically useful aptamers, particularly for cancer biomarkers and molecules involved in cancer proliferation, metastasis, and immune suppression. Recently a pair of 2′-fluoro-pyrimidine RNA aptamers with affinity for CA125 were reported [26]. These modified RNA aptamers were selected using a magnetic bead purification strategy on His-tagged CA125, with His-tagged VEGF used for counter-selection. Our future work will involve a comparison of the DNA aptamers reported here with these new RNA aptamers, and the evaluation of their possible synergistic use in the creation of novel assays for the detection of CA125. The availability of multiple aptamers with affinity for this clinically important target opens the possibility of developing a sandwich-format assay, with its inherent advantages of specificity and amplification.

## Figures and Tables

**Table 1:** Characteristics of data resulting from Illumina sequencing. R0 is the unselected random DNA library; other numbers designate the SELEX round and whether the DNA was bound to CA125 (+) or was not bound (−).

SELEX round	DNA sequenced	Number of reads	Sequence duplication
R0	Library	7.3 × 10^6^	15.24%
R1+	Bound	6.4 × 10^6^	16.94%
R1−	Unbound	7.6 × 10^6^	15.78%
R2+	Bound	5.7 × 10^6^	45.24%
R2−	Unbound	10.8 × 10^6^	18.00%
R3+	Bound	4.3 × 10^6^	78.71%
R3−	Unbound	4.1 × 10^6^	37.94%
R4+	Bound	7.4 × 10^6^	85.04%
R4−	Unbound	5.4 × 10^6^	82.62%

**Table 2:** CA125 aptamer candidates chosen for in vitro analysis. Aptamers candidates were named based on their enrichment rank; for example, CA125_1 is the sequence that was most enriched in Round 4 relative to the unselected library (Round 0). Note that CA125_2 is a 26 mer; indels of plus or minus one base were tolerated in our bioinformatics analysis. n/a: not available.

ID	Enrichment	Sequence	CompScore	Sequence cluster
CA125_1	844	ACTAGCTCCGATCTTTCTTATCTAC	8.13	n/a
CA125_2	224	CACTCTTTCATTTTATTTATAATTAT	7.13	n/a
CA125_3	290	TTCAATATTACTTATCTTTTTTTTT	6.92	3
CA125_12	124	TGCCTTATTACTCTCTCCTGTTAAC	6.62	1

**Table 3:** Proportions of the four nucleobases in the CA125 aptamer candidates chosen for in vitro characterization.

ID	% G	% A	% T	% C	% (G+C)	% (A+T)
CA125_1	8	20	40	32	40	60
CA125_2	0	27	58	15	15	85
CA125_3	0	20	68	12	12	88
CA125_12	8	16	44	32	40	60
